# LncRNA HULC Polymorphism Is Associated With Recurrent Spontaneous Abortion Susceptibility in the Southern Chinese Population

**DOI:** 10.3389/fgene.2019.00918

**Published:** 2019-10-04

**Authors:** Zhenzhen Fang, Yanfang Yang, Yufen Xu, Hanran Mai, Wanqi Zheng, Lei Pi, Lanyan Fu, Huazhong Zhou, Yaqian Tan, Di Che, Xiaoqiong Gu

**Affiliations:** ^1^Program of Molecular Medicine, Guangzhou Women and Children’s Hospital, Zhongshan School of Medicine, Sun Yat-Sen University, Guangzhou, China; ^2^Department of Prenatal Diagnosis, Maoming People’s Hospital, Maoming, China; ^3^Department of Clinical Biological Resource Bank, Guangzhou Institute of Pediatrics, Guangzhou Women and Children’s Medical Center, Guangzhou Medical University, Guangzhou, China; ^4^Department of Clinical Lab, Guangzhou Women and Children’s Medical Center, Guangzhou Medical University, Guangzhou, China; ^5^Department of Blood Transfusion, Guangzhou Women and Children’s Medical Center, Guangzhou Medical University, Guangzhou, China

**Keywords:** recurrent spontaneous abortion, HULC, susceptibility, polymorphism, rs7770772

## Abstract

Previous studies have revealed that genetic variation in genes that regulate cell migration might be associated with susceptibility to recurrent spontaneous abortion. HULC regulates the migration of a variety of cells, and genetic polymorphisms of HULC are associated with susceptibility to a variety of diseases, but their association with susceptibility to recurrent spontaneous abortion has not been reported. This study included 610 cases of recurrent spontaneous abortion and 817 normal controls, and the polymorphisms of the four SNPs were genotyped using the TaqMan method. Odds ratios (ORs) and 95% confidence intervals (CIs) were calculated to assess the associations between selected SNPs and susceptibility to recurrent spontaneous abortion. Our results showed that three SNPs were significantly associated with a reduced risk of recurrent spontaneous abortion: rs1041279 (GG vs. GC/CC: adjusted OR = 0.745, 95% CI = 0.559–0.993, P = 0.0445), rs7770772 (GC/CC vs. GG: adjusted OR = 0.757, 95% CI = 0.606–0.946, P = 0.0143), and rs17144343 (AA/GA vs GG adjusted OR = 0.526, 95% CI = 0.366–0.755, P = 0.0005). Individuals with one to four genotypes showed a reduced risk of recurrent spontaneous abortion (adjusted OR = 0.749, 95% CI = 0.598–0.939, P = 0.0123). This cumulative effect on protection increased with increases in the observed number of genotypes (adjusted OR = 0.727, 95% CI = 0.625–0.846, ptrend < 0.0001). Our study suggests that HULC might be a biomarker for risk for recurrent spontaneous abortion, but larger sample studies are needed to verify this result.

## Introduction

Recurrent spontaneous abortion is a pregnancy complication that occurs in approximately 5% of fertile couples (Rai and Regan, 2006). At present, the pathogenesis of recurrent spontaneous abortion is not fully understood, but it is know that several factors, such as chromosomal abnormalities, anatomical defects, thrombosis, immune factors, endocrine dysfunction, malnutrition, and environmental factors, are associated with the development of recurrent spontaneous abortion (Regan et al., 1989; Saravelos and Regan, 2014; Garrido-Gimenez and Alijotas-Reig, 2015; Kaur and Gupta, 2016; Pereza et al., 2017). With the development of genetic testing technology, the polymorphism of many genes was recently confirmed to be associated with susceptibility to recurrent spontaneous abortion, and some gene polymorphisms, such as KDR gene polymorphism, CTLA4 genetic polymorphism and MTHFR polymorphism, might predict the risk of recurrent spontaneous abortion (Honarvar et al., 2016; Li et al., 2018; Zhu et al., 2018). Abnormal immune reactions and dysfunction of trophoblast infiltration might lead to recurrent spontaneous abortion (Pearson, 2002; Sun and Zhang, 2017). Recent studies have revealed that some genetic polymorphisms related to immunity-regulation pathways and the regulation of cell motility, such as polymorphisms in IL-1β, p53, IGF-2, and PAI 1, are becoming increasingly important in recurrent spontaneous abortion (Honarvar et al., 2016; Li et al., 2018; Zhu et al., 2018). A recent study conducted by our research group confirmed that polymorphisms in the lncRNAs MALAT1 and CCAT2, which are involved in the regulation of immunity and cell motility, are associated with susceptibility to recurrent spontaneous abortion (Che et al., 2019a; Che et al., 2019b). Therefore, it is important to explore whether polymorphisms in lncRNAs involved in the regulation of immunity and cell motility are associated with susceptibility to recurrent spontaneous abortion, and the results would improve the understanding of the etiology of recurrent spontaneous abortion.

lncRNAs, which are RNA molecules with lengths greater than 200 nucleotides that are not translated into proteins, regulate gene expression in a variety of ways and play an important role in many human diseases (Shi et al., 2013). The lncRNA highly upregulated in liver cancer (HULC) is overexpressed in hepatocellular carcinoma and associated with multiple diseases, including tumor growth and angiogenesis, and the regulation of cell proliferation, migration and invasion (Cui et al., 2015; Lu et al., 2016; Kong and Wang, 2018). In recent years, an increasing number of studies have found that HULC polymorphisms are associated with susceptibility to various diseases, such as hepatocellular cancer and colorectal cancer (Shaker et al., 2017; Wang et al., 2018). A recent study revealed that genetic variations in the lncRNAs HULC and MALAT1 are associated with decreased susceptibility to hepatocellular carcinoma (in persistent carriers of HBV), and these lncRNAs thus constitute two potential diagnostic biomarkers for hepatocellular carcinoma (Motawi et al., 2019). Moreover, another study found that HULC cooperates with MALAT1 to aggravate the growth of liver cancer stem cells (Wu et al., 2016). A study conducted by Wang et al. confirmed that MALAT1 is downregulated in patients with recurrent spontaneous abortion, and this downregulated expression is one of the factors leading to the pathogenesis of recurrent spontaneous abortion (Wang et al., 2018). Our previous research also found that MALAT1 polymorphism is associated with susceptibility to recurrent spontaneous abortion (Che et al., 2019b). Similar to the findings obtained for lncRNA MALAT1, many studies have found that the lncRNA HULC is associated with inflammation and cell migration and invasion (Wang et al., 2016; Li et al., 2018). These studies suggest that lncRNA *HULC* gene polymorphisms might be associated with recurrent miscarriage. Therefore, we hypothesized that HULC polymorphisms might be associated with susceptibility to recurrent spontaneous abortion, but the effects of HULC polymorphisms in patients with susceptibility to recurrent spontaneous abortion have not been studied. Therefore, in this case–control study with 610 hospital-based cases and 817 controls, we aimed to determine the relationship between *HULC* gene polymorphisms (rs1041279, rs7770772, rs1328868, and rs1714434) and susceptibility to recurrent spontaneous abortion in southern China.

## Materials and Methods

### Study Subjects

All the participants included in this study were of Chinese Han descent. The control group was age-frequency-matched to the case group. From June 2017 to June 2019, a total of 610 recurrent spontaneous abortion patients and 817 healthy controls were recruited at the Guangzhou Women and Children Medical Center. Recurrent spontaneous abortion was diagnosed as more than two spontaneous abortions with the same husband (unknown cause, 5–20 weeks gestation), and the control women had at least two normal pregnancies and no history of miscarriage. Couples with chromosomal abnormalities were excluded from the case group. None of the patients in the case and control groups had metabolic disorders, autoimmune diseases, hypertension, endocrine disorders, arterial or venous thrombosis, uterine abnormalities, liver or kidney dysfunction, or embryonic chromosomal abnormalities.

The medical ethics committee of the Guangzhou Women and Children Medical Center approved the study. All the subjects who participated in the study provided signed informed consent (Guangzhou, China, 2018022202).

### SNP Genotyping and DNA Extraction

Peripheral blood was collected from the vein of each subject and placed in a tube containing EDTA. Genomic DNA samples were extracted from 200 μl of the samples of peripheral blood leukocytes obtained from all the participants using a DNA extraction kit (Tiangen, Beijing, China) according to the manufacturer’s instructions. We selected the Chinese Han Beijing (CHB) population and used the 1000G data (http://www.internationalgenome.org/home) to select SNPs of the *HULC* gene using the following three criteria: (1) the minor allele frequency (MAF) in the CHB population reported in HapMap was not less than 0.05; (2) TagSNP was selected based on pairwise linkage disequilibrium (LD) information to maximize representation (r^2^ > 0.8); and (3) the selected upstream and downstream regions of the *HULC* gene were expanded by 2,000 bp. The four candidate SNPs (rs1041279, rs7770772, rs1328868, and rs1714434) were genotyped on an ABI Q6 instrument (Thermo Fisher Scientific, USA) using a TaqMan real-time polymerase chain reaction protocol. And Linkage disequilibrium (LD) between four candidate SNPs are shown in **Supplementary Table S1**. The strength of linkage disequilibrium (LD) with the two SNPs was calculated by the online SHEsis software (https://analysis.bio-x.cn) (Shi and He, 2005). A specific fluorescent probe for genotyping was purchased from ABI (Thermo Fisher Scientific, USA). Two blank (water) controls in each 384-well plate were used for quality control. We also randomly selected 10% of the samples for repeated measurements, and the results were consistent.

### Statistical Analysis

The differences in the demographic characteristics and genotype frequencies of the SNPs between cases and controls were assessed using the χ2 test and Student’s t-test. Odds ratios (ORs) and their 95% confidence intervals (CIs) were calculated by logistic regression analysis to estimate the association between the genotypes and risk of recurrent spontaneous abortion susceptibility. The Hardy-Weinberg equilibrium (HWE) of the control group was calculated using the goodness of fit χ2 test. After adjusting for age, the adjusted OR was calculated through multivariate unconditional logistic regression. Subgroup analyses after stratification by age and number of abortions were also performed. All statistical analyses were performed using SAS statistical analysis software (version 9.4; SAS Institute, Cary, NC, USA). All tests were two-sided, and the statistical significance criterion was set to P < 0.05.

## Results

### Population Characteristics

We recruited 817 healthy controls and 610 patients with recurrent spontaneous abortions aged 20 to 49 years and 20 to 46 years, respectively, for inclusion in this study (**Table 1**). No significant differences in age were found between the control group and the patients with recurrent spontaneous abortion (32.81 ± 5.09 vs 32.43 ± 5.41 years, P = 0.1796). Approximately 59.18% of the patients with recurrent spontaneous abortion experienced two or three spontaneous abortions, and 40.82% of the patients with recurrent spontaneous abortion experienced four or more spontaneous abortions.

**Table 1:** Frequency distribution of selected characteristics in the group of patients with recurrent miscarriages and the control group.

Variables	Cases (*n* = 610)		Controls (*n* = 817)		*P* ^a^
	No.	%	No.	%	
Age range, year	20–46		20–49		
Mean ± SD	32.43 ± 5.41		32.81 ± 5.09		0.1796
<35	403	66.07	532	64.96	
35–40	150	24.59	217	26.5	
>40	57	9.34	70	8.55	
No. of abortion/%					
2–3	361	59.18			
≥4	249	40.82			

a The distributions between the cases and controls were examined using the two-sided χ2 test. The data are presented as the means ± SDs, and the p values were obtained using Student’s t test.

### Association Analysis

The allele and genotype frequencies of the four SNPs were selected, and their relationship to risk of recurrent spontaneous abortion is summarized in **Table 2**. All four selected SNPs were in Hardy-Weinberg equilibrium (HWE) in the control group (rs1041279, P = 0.202; rs7770772, P = 0.258; rs1328868, P = 0.595; and rs17144343, P = 0.124). Compared with the rs1041279 CC/GC genotype, the variant GG genotype was significantly associated with a reduced risk of recurrent spontaneous abortion (GG vs CC: adjusted OR = 0.745, 95% CI = 0.559–0.993, P = 0.0445). Moreover, the rs770772 GC/CC variant was found to be associated with susceptibility to recurrent spontaneous abortion (GC/CC vs GG: adjusted OR = 0.757, 95% CI = 0.606–0.946, P = 0.0143). We also found that the rs17144343 A allele carriers had a reduced risk of recurrent spontaneous abortion (GA vs GG: adjusted OR = 0.489, 95% CI = 0.334–0.717, P = 0.0002; GA/AA vs GG adjusted OR = 0.526, 95% CI = 0.366–0.755, P = 0.0005). In addition, the following genotypes were used for the calculations: rs1041279 GG, rs7770772 GC/CC, rs1328868 TT and rs17144343 GA/AA. We found that the individuals with one to four genotypes exhibited a reduced risk of recurrent spontaneous abortion (adjusted OR = 0.749, 95% CI = 0.598–0.939, P = 0.0123). This cumulative effect depended on the number of genotypes, and the risk of recurrent abortion significantly decreased with increases in the number of observed genotypes (adjusted OR = 0.727 95% CI = 0.625–0.846 for four genotypes; p_trend_ < 0.0001).

**Table 2:** Genotype and allele frequencies of the *HULC* gene in RSA patients and controls.

Genotype/allele	RSA (N = 610)	Controls (N = 817)	*P* ^a^	OR(95% CI)	*P* ^b^	Adjusted OR (95% CI)	*P* *^c^*
**HULC/rs1041279 C > G (HWE = 0.202) Loc. Promoter**							
CC	175(28.69)	246(30.04)	0.0644^d^	1.00	/	1.00	/
GC	347(56.89)	422(51.53)	/	1.156(0.909–1.470)	0.2374	1.148(0.902–1.460)	0.2617
GG	88(14.43)	151(18.44)	/	0.819(0.591–1.136)	0.2315	0.815(0.588–1.130)	0.2199
GC/GG	435(71.31)	573(69.96)	0.5800^e^	1.067(0.847–1.344)	0.5811	1.060(0.842–1.335)	0.6198
GC/CC	522(85.57)	668(81.56)		1.00	/	1.00	/
GG	88(14.43)	151(18.44)	**0.0433^f^**	**0.746(0.560–0.993)**	**0.0449**	**0.745(0.559–0.993)**	**0.0445**
**HULC/rs7770772 G > C (HWE = 0.258) Loc. Promoter**							
GG	421(69.02)	513(62.64)	**0.0392^d^**	1.00	/	1.00	/
GC	169(27.70)	277(33.82)	/	**0.743(0.590–0.936)**	**0.0118**	**0.749(0.594–0.943)**	**0.0141**
CC	20(3.28)	29(3.54)	/	0.840(0.469–1.507)	0.5595	0.834 (0.465–1.496)	0.5423
GC/CC	189(30.98)	306(37.36)	**0.0120^e^**	**0.753(0.603–0.940)**	**0.0123**	**0.757(0.606–0.946)**	**0.0143**
GC/GG	590(96.72)	790(96.46)		1.00	/	1.00	/
CC	20(3.28)	29(3.54)	0.7872^f^	0.924(0.518–1.649)	0.7889	0.914 (0.511–1.632)	0.7601
**HULC/rs1328868 C > T (HWE = 0.595) Loc. Exon**							
CC	269(44.10)	381(46.52)	0.6609^d^	1.00	/	1.00	/
CT	280(45.90)	360(43.96)	/	1.102(0.883–1.374)	0.3903	1.104(0.885–1.378)	0.3793
TT	61(10.00)	78(9.52)	/	1.108(0.765–1.603)	0.5876	1.101(0.761–1.594)	0.6086
CT/TT	341(55.90)	438(53.48)	0.3630^e^	1.103(0.893–1.361)	0.3632	1.104(0.894–1.363)	0.3591
CT/CC	549(90.00)	741(90.48)		1.00	/	1.00	/
TT	61(10.00)	78(9.52)	0.7641^f^	1.056(0.742–1.502)	0.7631	1.048(0.737–1.492)	0.7929
**HULC/rs17144343 G > A (HWE = 0.124) Loc. 3′-UTR**							
GG	564(92.46)	710(86.69)	**0.0008^d^**	1.00	/	1.00	/
GA	40(6.56)	102(12.45)	/	**0.494(0.337–0.724)**	**0.0003**	**0.489(0.334–0.717)**	**0.0002**
AA	6(0.98)	7(0.85)	/	1.079(0.361–3.229)	0.8918	1.059(0.354–3.171)	0.9183
GA/AA	46(7.54)	109(13.31)	**0.0004^e^**	**0.531(0.370–0.763)**	**0.0006**	**0.526(0.366–0.755)**	**0.0005**
GA/GG	604(99.02)	812(99.15)		1.00	/	1.00	/
AA	6(0.98)	7(0.85)	0.8002^f^	1.154(0.386–3.450)	0.7981	1.133(0.379–3.392)	0.8228
**Combined effect of genotypes^g^**							
0	291(47.70)	316(38.58)		1.00	/	1.00	/
1	255(41.80)	370(45.18)		**0.748(0.597–0.938)**	**0.0117**	**0.749(0.598–0.939)**	**0.0123**
2	63(10.33)	125(15.26)		**0.547(0.389–0.771)**	**0.0006**	**0.545(0.387–0.768)**	**0.0005**
3	1(0.16)	8(0.98)		0.136(0.017–1.092)	0.0605	0.137(0.017–1.103)	0.0618
Trend			**0.0003**	**0.728(0.625–0.847)**	** <.0001**	**0.727(0.625–0.846)**	** <.0001**
0	291(47.70)	316(38.58)	0.0006	1.00	/	1.00	/
1–4	319(52.30)	503(61.42)		0.748(0.597–0.938)	**0.0117**	**0.749(0.598–0.939)**	**0.0123**

^a^The genotype distributions between the cases and controls were examined using the two-sided χ2 test.

^b^Unadjusted for age in the logistic regression models.

^c^Adjusted for age in the logistic regression models.

^d^For additive genetic models.

^e^For dominant genetic models.

^f^For recessive genetic models.

^g^The genotypes used for the calculation were rs1041279 GG + rs7770772 GC/CC + rs1328868 TT + rs17144343 GA/AA.

Bold values are statistically significant (P < 0.05).

### Stratified Analysis

We further explored the relationship between *HULC* gene polymorphism (rs1041279, rs7770772, and rs17144343) and the combined effects of genotypes with recurrent spontaneous abortion susceptibility through stratification by age and number of abortions (shown in **Table 3**). Compared with the CC/CG genotype of rs1041279, the variant GG genotype was more protective for women aged 35–40 years (OR = 0.475, 95% CI = 0.261–0.866, P = 0.015). Compared with the GG genotype of rs7770772, the variant GC/CC genotype was more protective for women aged less than 35 years (OR = 0.682, 95% CI = 0.516–0.901, P = 0.007) and more protective for the patients who had at least four miscarriages (adjusted OR = 0.682, 95% CI = 0.501–0.928, P = 0.015). Compared with the GG genotype of rs17144343, the variant GA/AA genotype was more protective for women aged less than 40 years and more protective for the patients who had at least four miscarriages (adjusted OR = 0.449, 95% CI = 0.260–0.774, P = 0.004). In addition, a comprehensive analysis showed that one to four genotypes were more highly associated with a reduced risk of recurrent spontaneous abortion in women aged less than 35 years (OR = 0.686, 95% CI = 0.528–0.891, P = 0.005) and patients with at least four miscarriages (adjusted OR = 0.586, 95% CI = 0.440–0.780, P = 0.0003) compared with those with no genotypes. The genotypes shows no significant association in the other stratified analyses. The false-positive report probability (FPRP) values for the positive results of the *HULC* gene were shown in **Table 4**. The predicted value of the false positive report was 0.2, and the prior probability was 0.1. Most of the statistically significant findings were also significant except for the rs1041279. Compared to the rs7770772 GG genotype, the rs7770772 GC genotype (FPRP = 0.116) and GC/CC genotypes (FPRP = 0.116) were reduced, the recurrent spontaneous abortion risk is still credible. Compared to the 17144343 GG genotype, the rs17144343 GA genotype (FPRP = 0.032) and GA/AA genotypes (FPRP = 0.04) were reduced, the recurrent spontaneous abortion risk is still credible.

**Table 3:** Stratification analysis of HULC polymorphism in RSA.

Variable	rs1041279 (cases/controls)		P	OR (95% CI)	P	rs7770772 (cases/controls)		P	OR (95% CI)	P	rs17144343 (cases/controls)		P	OR (95% CI)	P	Combined (cases/controls)		P	OR (95% CI)	P
	CC/CG	GG				GG	GC/CC				GG	GA/AA				0	1–4			
Age																				
<35	342/442	61/90	0.463	0.876 (0.615–1.248)	0.464	**289/337**	**114/195**	**0.007**	**0.682** **(0.516–0.901)**	**0.007**	369/457	34/75	**0.007**	**0.562** **(0.366–0.861)**	**0.008**	198/212	205/320	**0.005**	**0.686** **(0.528–0.891)**	**0.005**
35–40	133/171	17/46	**0.012**	**0.475** **(0.261–0.866)**	**0.015**	95/133	55/84	0.691	0.917 (0.596–1.409)	0.692	141/188	9/29	**0.019**	**0.414** **(0.190–0.902)**	**0.026**	66/77	84/140	0.101	0.700 (0.457–1.072)	0.101
>40	47/55	10/15	0.583	0.780 (0.320–1.899)	0.585	37/43	20/27	0.686	0.861 (0.416–1.780)	0.686	37/43	3/5	0.662	0.722 (0.165–3.161)	0.666	27/27	30/43	0.319	0.698 (0.344–1.417)	0.319
No. of abortion/%				Adjust OR (95% CI)	P^a^				Adjust OR (95% CI)	P^a^				Adjust OR (95% CI)	P^a^				Adjust OR (95% CI )	P^a^
2–3	306/668	55/151	0.177	0.800 (0.570–1.123)	0.197	244/513	117/306	0.101	0.804 (0.619–1.045)	0.102	331/710	30/109	**0.011**	**0.581** **(0.379–0.890)**	**0.012**	163/316	198/503	0.035	**0.763** **(0.593–0.981)**	**0.035**
≥4	216/668	33/151	0.052	0.665 (0.442–1.000)	0.05	177/513	72/306	**0.014**	**0.68** **2(0.501–0.928)**	**0.015**	**233/710**	**16/109**	**0.002**	**0.449** **(0.260–0.774)**	**0.004**	**128/316**	**121/503**	**0.000**	**0.586** **(0.440–0.780)**	**0.003**

The genotypes used for the calculation were rs1041279 GG + rs7770772 GC/CC + rs1328868 TT + rs17144343 GA/AA.

^a^Adjusted for age. Bold values are statistically significant (P <0.05).

**Table 4:** False-positive report probability values for associations between recurrent spontaneous abortion risk and genotypes of HULC polymorphisms.

Genotype/Allele	OR (95% CI)	p-value^a^	Statistical power^b^	prior probability				
				0.25	0.1	0.01	0.001	0.0001
**HULC/rs1041279 C > G**								
GC/CC Vs. GG	0.746(0.560–0.993)	0.0449	0.756	0.151	0.348	0.855	0.983	0.998
**HULC/rs7770772 G> C **								
GC Vs. GG	0.743(0.590–0.936)	0.0118	0.811	0.042	**0.116**	0.59	0.936	0.993
GC/CC Vs. GG	0.753(0.603–0.940)	0.0123	0.841	0.042	**0.116**	0.591	0.936	0.993
**HULC/rs17144343 G > A **								
GA Vs. GG	0.494(0.337–0.724)	0.0003	0.082	0.011	**0.032**	0.266	0.785	0.973
GA/AA Vs. GG	0.531(0.370–0.763)	0.0006	0.129	0.014	**0.04**	0.316	0.823	0.979
**Combined effect of genotypes **								
1 Vs. 0	0.748(0.597–0.938)	0.0117	0.873	0.039	**0.108**	0.57	0.93	0.993
2 Vs. 0	0.547(0.389–0.771)	0.0006	0.163	0.011	**0.032**	0.267	0.786	0.974
1–4 Vs. 0	0.748(0.597–0.938)	0.0117	0.882	0.038	**0.107**	0.568	0.93	0.993

^a^χ2 test was used to calculate the genotype frequency distributions.

^b^Calculated the statistical power using the number of observations and the OR and p values statistically significant values are shown in bold (P < 0.05).

## Discussion

In this study, we screened four SNPs in the *HULC* gene and found that the variant genotypes of rs1041279 C > G, rs7770772 G > C and rs17144343 G > A were associated with a reduced risk of recurrent spontaneous abortion. The protective effect was most pronounced in women younger than 35 years and the patients who had at least four miscarriages. This study constitutes the first investigation of the relationship between *HULC* gene polymorphisms and susceptibility to recurrent spontaneous abortion. Our study suggests that HULC might be a biomarker for risk of recurrent spontaneous abortion. The sample size included in this study is relatively small, and studies with larger sample size are needed to verify this result.

An increased number of studies confirm that the lncRNA HULC plays an important role in many diseases. HULC affects the migration and invasion of cells, including mesenchymal stem cells, osteosarcoma cells, and gastric cancer cells (Kong and Wang, 2018; Li et al., 2018; Ma and Ding, 2018). In addition, HULC can also regulate epithelial-to-mesenchymal transition (EMT) process in oral squamous cell carcinoma (Su et al., 2019). Previous studies have also found that the invasion and migration functions of extravillous trophoblast cells are closely related to the occurrence of recurrent spontaneous abortion (Li et al., 2017; Windsperger et al., 2017). Trophoblastic cells undergo EMT and become mesenchymal trophoblasts, which is the prerequisite for their invasion and migration (Tantbirojn et al., 2008; Nakaya and Sheng, 2013; Li et al., 2014; Miner et al., 2016; Zong et al., 2016; Zhou et al., 2017). These studies suggest that HULC might play an important role in the pathogenesis of recurrent spontaneous abortion. Recently, Fenoglio et al. confirmed that lncRNA HULC expression is downregulated in multiple sclerosis patients (Fenoglio et al., 2018). A study conducted by Shaker et al. revealed that the lncRNA HULC is abnormally expressed in colorectal cancer patients and that this abnormal expression is associated with its polymorphism (Shaker et al., 2017). Therefore, *HULC* gene polymorphisms might be associated with susceptibility to recurrent abortion. In this study, we selected the four SNPs (rs1041279, rs7770772, rs1328868, and rs17144343) that were more likely to be associated with recurrent spontaneous abortion. The rs1041279 C > G and rs7770772 G > C polymorphisms are located in the *HULC* gene promoter, rs1328868 C > T is located in an exon, and rs17144343 G > A is located in the 3′-UTR. Our study suggests that three SNPs in the lncRNA *HULC* gene (rs1041279 C > G, rs7770772 G > C, and rs17144343 G > A) reduce susceptibility to recurrent spontaneous abortion. In addition, the cumulative protective effect depends on the number of genotypes, and the risk of recurrent abortion is significantly reduced with increases in the number of observed genotypes. Promoter polymorphisms might have important functions in regulating the expression of mature lncRNAs and some transcription factors (Bongiorni et al., 2014). However, Wang et al. found that rs1041279 in the *HULC* gene is associated with increased risk for hepatocellular cancer and suggested that this polymorphism might be a biomarker for hepatocellular cancer risk (Wang et al., 2018). Unfortunately, these researchers did not assess whether rs17144343 is associated with susceptibility to hepatocellular cancer risk (Wang et al., 2018). Therefore, rs1041279, rs7770772, and rs17144343 might be SNPs with multiple functions that could have the potential to predict the risk of recurrent spontaneous abortion.

A study conducted by Wu et al. found that MALAT1 combined with HULC promotes the malignant progression of liver cancer stem cells through microsatellite instability and telomere alteration (Wu et al., 2016). Moreover, Fan et al. suggested that MALAT1 is an important mediator of the TGF-β-induced EMT by promoting bladder cancer metastasis (Fan et al., 2014). Studies have also found that HULC regulates EMT in a variety of cancers, such as gastric cancer and hepatocellular carcinoma (Zhao et al., 2014; Li et al., 2016). Furthermore, HULC reduces the expression of EMT-related factors in bone neoplasm cells (Zhang et al., 2018). The genetic polymorphisms of HULC and MALAT1 are associated with susceptibility to recurrent spontaneous abortion. In addition, MALAT1 is decreased in patients who experience complicated recurrent spontaneous abortions (Wang et al., 2018). Although we did not detect the expression of HULC, we hypothesized that HULC polymorphisms might regulate the expression of HULC. Thus, HULC combined with MALAT1 regulates the EMT signaling pathway to protect against recurrent abortion, but this hypothesis requires further experimental verification.

Numerous studies have shown that advanced age and number of previous abortions are risk factors for miscarriage: women over 40 years of age are five times more likely to have a miscarriage than women between the ages of 31 and 35. Other studies have confirmed that the number of previous abortions is related to the risk of miscarriage (van Kooij et al., 1996; Nybo Andersen et al., 2000; Ogasawara et al., 2000; Agenor and Bhattacharya, 2015). In our study, combination of the *HULC* gene genotypes revealed that the protective effect was most pronounced in women younger than 35 years and patients who had at least four miscarriages, which might explain the reduction in recurrent spontaneous abortion in women younger than 35 years compared with women older than 35 years. However, this specific molecular mechanism needs to be further studied. In conclusion, our findings further demonstrate the important role of the *HULC* gene in the development of recurrent spontaneous abortion.

This study constitutes the first exploration of the relationship between HULC polymorphisms and susceptibility to recurrent spontaneous abortion. Many limitations of this study should be noted. First, the sample size of the case and control groups in this study was relatively small, which hinders the statistical effectiveness of the study. Second, in this study, we focused on four HULC SNPs. Moreover, the expression of HULC was not detected in this study, and thus, additional SNPs that might affect the expression and function of HULC will be studied in the future. Third, because this study is retrospective in nature, some important information might be associated with factors related to recurrent spontaneous abortion (e.g., lifestyle, smoking, and drinking). Fourth, we only included women in southern China in this study. These findings cannot be extended from to another race until additional validation studies of more women with different genetic backgrounds are conducted.

In conclusion, this study suggests that the rs1041279 GG allele, the rs7770772 GC/CC alleles, and the rs17144343 GA/AA allele of the *HULC* gene are associated with decreased susceptibility to recurrent spontaneous abortion and protect patients against abortion. In addition, the protective effect is most pronounced in women younger than 35 years and patients who have had at least four miscarriages. However, future research should include a larger sample size and more experiments to further explore the role of the *HULC* gene in defining the susceptibility to recurrent spontaneous abortion.

## Data Availability Statement

The raw data supporting the conclusions of this manuscript will be made available by the authors, without undue reservation, to any qualified researcher.

## Ethics Statement

The studies involving human participants were reviewed and approved by The medical ethics committee of the Guangzhou Women and Children Medical Center. The patients/participants provided their written informed consent to participate in this study.

## Author Contributions

All the authors contributed significantly to this work. DC, YX, YY and ZF devised the research plan. The data were analyzed by ZF and HM. DC wrote the manuscript, and YT, WZ and HZ performed the experiments. LP and LF designed the experimental methods, and XG modified and polished the manuscript. All the authors support the publication of the manuscript.

## Funding

This study was supported by the Guangdong Natural Science Fund, China (grant number 2016A030313836); the Guangzhou Medical and Health Technology Projects, China (grant number 20171A011260); and the Guangzhou Science and Technology Program Project, China (grant numbers 201607010011 and 201804010035).

## Conflict of Interest

The authors declare that the research was conducted in the absence of any commercial or financial relationships that could be construed as a potential conflict of interest.
