# Supplementary material for: LncRNA HULC Polymorphism Is Associated With Recurrent Spontaneous Abortion Susceptibility in the Southern Chinese Population
Source: Front Genet. 2019 Oct 4;10:918. doi: 10.3389/fgene.2019.00918 (PMC6788392; doi:10.3389/fgene.2019.00918)
Supplement: Supplementary file 1 [file Table_1.docx]

**Table S1**

Linkage disequilibrium between four SNPs of lncRNA HULC gene

| **control** | | | |  | **case** | | | |
| --- | --- | --- | --- | --- | --- | --- | --- | --- |
| D' | rs7770772 | rs1328868 | rs17144343 |  | **D'** | rs7770772 | rs1328868 | rs17144343 |
| rs1041279 | 0.922 | 0.885 | 0.562 |  | rs1041279 | 0.94 | 0.899 | 0.824 |
| rs7770772 | - | 0.908 | 0.605 |  | rs7770772 | - | 0.938 | 0.644 |
| rs1328868 | - | - | 0.152 |  | rs1328868 | - | - | 0.997 |
|  |  |  |  |  |  |  |  |  |
| **r^2^** | rs7770772 | rs1328868 | rs17144343 |  | **r^2^** | rs7770772 | rs1328868 | rs17144343 |
| rs1041279 | 0.173 | 0.455 | 0.03 |  | rs1041279 | 0.137 | 0.53 | 0.04 |
| rs7770772 | - | 0.097 | 0.007 |  | rs7770772 | - | 0.089 | 0.004 |
| rs1328868 | - | - | 0.001 |  | rs1328868 | - | - | 0.022 |
